# Effects of Host Plant on the Bacterial Community of the Leafhopper *Scaphoideus titanus*

**DOI:** 10.3390/insects16111144

**Published:** 2025-11-08

**Authors:** Andrea Arpellino, Aya M. A. Elsayed, Elena Gonella, Alberto Alma

**Affiliations:** Department of Agricultural, Forest and Food Sciences, University of Torino, 10095 Grugliasco, Italy; andrea.arpellino@unito.it (A.A.); aya.elsayed@unito.it (A.M.A.E.); alberto.alma@unito.it (A.A.)

**Keywords:** insect microbiome, *Karelsulcia*, *Cardinium*, non-core microbiome, flavescence dorée phytoplasma

## Abstract

**Simple Summary:**

Management of *Scaphoideus titanus* poses a significant challenge to viticulture in Europe, as this leafhopper is the main vector of Flavescence Dorée phytoplasma. An important constraint in *S. titanus* control is its frequent immigration into vineyards from surrounding uncultivated areas containing wild vines originating from American *Vitis* rootstock. Movement between wild and cultivated plants may influence the biology of the insect as well as its microbiome, possibly affecting the insect efficiency in transmitting phytoplasmas. However, this aspect has received little attention so far. In this study, we evaluated the effect of feeding on different *Vitis* species on the bacterial communities associated with *S. titanus* immatures. A general reduction in the bacterial diversity was observed throughout insect development, and a variability of the non-dominant bacterial community was found according to the plant species. These results indicate a change in the microbiome composition of nymphs, which may have consequences both for the insect performance and the bacterial exchange with the plant at the adult stage. Understanding the dynamics of plant–insect–microbiome interactions is important because they may affect the biology and behavior of *S. titanus*, and, ultimately, phytoplasma epidemiology.

**Abstract:**

The Nearctic leafhopper *Scaphoideus titanus* is the primary vector of Flavescence Dorée, a severe grapevine disease in Europe. This insect can complete its life cycle on both cultivated *Vitis vinifera* and American *Vitis* species, including rootstock-derived plants that have gone wild. While the movement of *S. titanus* between wild and cultivated vines is well documented, its biological implications remain unclear, particularly regarding the role of the insect-associated microbiome. In this study, we investigated how rearing *S. titanus* nymphs on different host plants, including American *Vitis* and several *V. vinifera* cultivars, affects its bacterial community. 16S rRNA metabarcoding revealed that the bacterial microbiome was dominated by two obligate symbionts, namely ‘*Candidatus* Karelsulcia’ and ‘*Candidatus* Cardinium’, with moderate but significant differences in microbial diversity among host plants and developmental stages. When these dominant symbionts were excluded, variability in the remaining bacterial community increased, indicating a modulation of minor taxa according to the plant offered. These findings suggest that host plant species influence the microbiome structure, potentially affecting the insect performance and the microbial exchange between wild and cultivated vines in the field, contributing to disease dynamics.

## 1. Introduction

The order Hemiptera is one of the most diverse insect taxa, containing many of the most troublesome agricultural pests. This diversification is partly due to the ancient relationships that hemipterans have established with bacteria. Bacterial symbionts can manipulate the host behavior and phenotype, resulting in various host adaptations [1]. The microbiome of Hemiptera, especially across the Auchenorrhyncha suborder, comprises few obligate bacteria (i.e., primary symbionts), which are mostly located intracellularly in specialized organs, as well as several facultative partners (i.e., secondary symbionts) [2]. Facultative symbionts may be located internally in several organs and body parts, or externally on the cuticle [3]. In addition to these, hemipterans, like all insects, harbor a variety of commensals and other occasional associates in their guts [4,5,6]. These latter groups play an important role in shaping the insect intraspecific microbiome variation, which in turn affects its ability to colonize and persist in an ecosystem (e.g., by influencing its plant host range, reproduction, and behavior) [7]. A major cause for intraspecific microbiome variability is the food source, as individuals feeding on different substrates are prone to ingesting and acquiring different microbial consortia [8]. Indeed, multiple studies have described the diet as an important factor shaping the insect microbiome in several orders, including Hemiptera, especially considering gut-associated microbes [9,10,11,12]. Plant-feeding Hemiptera may take advantage from the nutritional support provided by beneficial symbionts acquired during feeding, thereby extending their potential host range to plants that would otherwise be unable to support the insect development in the absence of these microbial partners [13]. In generalist insect species, the combination of these phenomena may eventually lead to host race differentiation or even sympatric speciation [7]. Furthermore, the effects of microbiome variations may be dynamically modulated, given the often-dramatic rearrangement of the microbiome that insects undergo during post-embryonic development due to tissue replacement and physiological changes [14].

The American leafhopper *Scaphoideus titanus* Ball (Hemiptera, Cicadellidae) poses a significant threat to European viticulture as it is the most efficient vector of Flavescence Dorée (FD) phytoplasma. The association between *S. titanus* and FD phytoplasma was established following the leafhopper introduction from the Nearctic region to the western Palearctic region in the 1950s, leading to widespread FD outbreaks across the main European wine-growing areas [15,16]. Since this novel association has started, FD phytoplasma has become an integral part of the facultative microbiome of *S. titanus*, colonizing several insect organs including midgut, hemolymph and salivary glands [15]. However, the infection with FD phytoplasma is not the only new microbial association that has been established in *S. titanus*. At least one other symbiont, namely ‘*Candidatus* Cardinium sp.’, (hereafter *Cardinium*) is thought to have been acquired after the leafhopper was introduced to Europe, as it is found exclusively in European populations, where it largely dominates the bacterial microbiome [17]. Another dominant bacterial endosymbiont of *S. titanus* is the primary symbiont ‘*Candidatus* Karelsulcia meulleri’ (hereafter *Karelsulcia*), which is found in all Auchenorrhyncha [2]. In addition to these two symbionts, the bacterial microbiome of *S. titanus* exhibits relatively low diversity [18]. This is consistent with the oligophagous nature of this species, as *S. titanus* can complete its life cycle exclusively on one genus, i.e., *Vitis* [15]. However, in European agroecosystems *S. titanus* can feed on different cultivars of cultivated grapevine or even on species other than *Vitis vinifera*, such as gone-wild vines deriving from American *Vitis* spp. used for rootstock [19,20,21]. Feeding on a different host source results in different fitness and feeding behaviors: cultivars that support the insect development more effectively also allow for a longer duration of the phloem feeding phase [22,23]. Variations in the endophytic bacterial community have been reported among grapevine cultivars [24] and between wild and domesticated vine species [25]. Therefore, the differential life traits of *S. titanus* may also correlate with different microbiome profiles acquired from the plant. Additionally, *S. titanus* has been found to inoculate grapevines with various microorganisms besides FD phytoplasma, thereby spreading potentially beneficial endophytes from plant to plant [26]. In this context, this study aimed at answering the following questions: (i) Does the development of *S. titanus* on different host plants affect the composition of their microbiome across the pre-adult stages? (ii) Are the potential changes related to dominant symbionts or the non-core microbiome? (iii) Does the possible variation in microbiome acquired by nymphs from different host plants have the potential to influence the endophyte community of other plants on which they feed as adults? Addressing these questions is important for increasing the knowledge of the relationship between *S. titanus* and *Vitis* spp. hosts in terms of shared microorganisms, which may in turn affect the multiple interactions occurring between the insect, the plant, and their microbiome, thereby determining the vector performance and competence for FD phytoplasma transmission.

## 2. Materials and Methods

### 2.1. Insect Rearing on Single Plant Sources

Between January and March 2022, two-year-old grapevine (*V. vinifera*) canes were collected during the winter pruning in several vineyards located in areas of the Piedmont region where a single cultivar is mainly grown. The sampling points were in the following municipalities: Albiano d’Ivrea (Torino Province, 45°4325′ N, 7°9676′ E), Erbaluce cultivar; Vaglio Serra (Asti Province, 44°7989′ N, 8°3409′ E), Barbera cultivar; Novi Ligure (Alessandria Province, 44°7427′ N, 8°7960′ E), Chardonnay cultivar. Where possible, vineyards with limited borders of non-vineyard land and that bordered other vineyards trained with the same cultivar were chosen. Beside vegetation composition, the vineyards were selected also based on monitoring data made available by the Phytosanitary Service of Piedmont, as high numbers of *S. titanus* had been caught there in previous years. Additionally, canes from wild *Vitis* spp. (i.e., *Vitis berlandieri* or gone-wild vines originating from American rootstocks) were collected in wild areas close to vineyards in Canale d’Alba (Cuneo Province, 44°8028′ N, 7°9810′ E). The canes from different areas and host plants were stored separately during the winter in outdoor rearing cages made of polyethylene and insect-proof nylon mesh (75 cm × 75 cm × 120 cm high) at the DISAFA laboratories. After budburst, two healthy potted grapevine plants were added to each cage to sustain the first instar nymphs after egg hatching. The potted plants were commercially purchased grafted vines with an American rootstock (S.O.4, *V. berlandieri* × *Vitis riparia*) and a scion made of a clone of the same cultivar as the original canes (Barbera: I–AT 84, Erbaluce: I–CVT TO 30, Chardonnay: SMA 123). The plants for the American *Vitis* group were the same rootstock without a scion.

The emergence of first instar nymphs was checked daily; emerging insects were attributed to *S. titanus* based on the distinctive traits indicated in [15]. Twenty *S. titanus* nymphs that hatched on the same day from each separate rearing were randomly collected using a fine brush to constitute one replicate. The insects belonging to each replicate were transferred in a climate chamber at 24 ± 1 °C, with a photoperiod of 16 h light and 8 h dark, into a Plexiglas cage containing one potted plant of the same type (i.e., cultivar clone or *Vitis* species). The nymphs were monitored daily until they either reached the adult stage or died. The trials were replicated five times.

### 2.2. Metabarcoding Analysis of Nymph Bacterial Community

#### 2.2.1. Insect Collection, DNA Extraction and Sequencing

A subset of insects from each separate rearing and replicate (up to five specimens per group) was collected for metabarcoding analysis at two distinct life stages (early nymph, I–II instar; late nymph, III–V instar), to represent separate timepoints of the development of immatures. In total, 87 *S. titanus* nymphs were analyzed, including 10 specimens reared on Barbera cultivar (4 early and 6 late nymphs), 20 on Chardonnay cultivar (6 early and 14 late nymphs), 11 on Erbaluce cultivar (5 early and 6 late nymphs), and 46 on American *Vitis* (21 early and 25 late nymphs). Each insect was individually preserved at −20 °C in absolute ethanol until submitted to DNA extraction, in order to exclude external components of the bacterial community. DNA extraction was done from single whole insects as described by Gonella et al. [27]. Briefly, insect tissues were disrupted in 550 µL of TE buffer (10 mM Tris-HCl, 1 mM EDTA, pH 8.0) using pestles, followed by lysozyme (10 mg·mL^−1^) and proteinase K (20 mg·mL^−1^) digestion, and SDS (10%) lysis. DNA was purified using NaCl (5 M) and CTAB buffer (15% CTAB, 0.7% NaCl), extracted sequentially with phenol–chloroform–isoamyl alcohol (25:24:1, pH 8.0) and chloroform–isoamyl alcohol (24:1, pH 8.0), then precipitated with pure isopropanol and 70% ethanol and resuspended in TE buffer. All reagents of extraction were purchased by Merck (Darmstadt, Germany). One negative control with DNA extraction reagents alone was included. After nucleic acids extraction, DNA samples were used for a metabarcoding approach to characterize the bacterial composition of their microbiome by sequencing the V3–V4 hypervariable regions of the 16S rRNA gene. Libraries were prepared with Herculase II Fusion DNA Polymerase and the Nextera XT Index V2 kit (Illumina, San Diego, CA, USA), based on manufacturer’s guidelines, with 341f/805r primer pair, targeting the 16S rRNA gene V3–V4 regions [28]. Paired-end sequences (301 pb) were obtained with the Illumina MiSeq platform. Library preparation and sequencing was performed by Macrogen, Inc. (Seoul, Republic of Korea).

#### 2.2.2. Sequence Analysis

Prior to analyzing the raw sequences, sequences with less than 100 bp were removed, using the ‘fastp’ pipeline [29]. Then the raw data were processed by using the QIIME2 pipeline (version 2024.10) [30]. Sequences were trimmed by 15 bp at the beginning of the forward and reverse reads. The forward reads were then truncated at 280 bp and the reverse reads were truncated at 230 bp using DADA2 pipeline in QIIME2 [31]. DADA2 was also used for the clustering and denoising of the raw reads to generate amplicon sequence variants (ASVs) [31]. Taxonomic classification was performed by using the SILVA database, version 138 [32]. Subsequently, ASVs belonging to the Archaea or Eukaryota domains and those related to mitochondria and chloroplasts were removed along with rare ASVs (i.e., singletons) and those from the negative control sample. ASVs that were unclassified at the genus level were further investigated by means of BLAST search (Nucleotide BLAST, version 2.14.1) against the NCBI database (with a 97% similarity threshold) in order to manually assign a genus. Two ASV tables were generated, one including all ASVs (named full microbiome), and the other after removal of the two dominant ASVs (named non-core microbiome).

To calculate bacterial diversity, rarefaction was initially performed to standardize the sequencing depth across samples, ensuring comparability by subsampling each sample to the same number of reads. Shannon, Simpson, Chao1 indices were then calculated using the ‘phyloseq’ package (version 1.55.0) [33]. A Kruskal–Wallis sum rank test was conducted, followed by pairwise comparisons (Dunn’s Kruskal–Wallis test) between treatments (i.e., host plant, developmental stage or their interaction) adjusted using the Bonferroni post hoc method (*p* < 0.05).

To assess beta diversity, feature tables were Hellinger-transformed and Bray–Curtis dissimilarity was calculated using the ‘vegan’ package (version 2.7-1) [34]. The results were visualized by Principal Coordinates Analysis (PCoA) plot. Permutational Multivariate Analysis of Variance (PERMANOVA) was performed to test the Bray–Curtis distance, fixed with 9999 permutations between treatments using ‘adonis2’ function of the vegan package [34]. To ensure that the potential differences were not due to group dispersion, a homogeneity of variances test (betadisper) was performed [35].

To evaluate the variation in the two dominant symbionts of European *S. titanus* populations, namely the primary symbiont *Karelsulcia* and the secondary symbiont *Cardinium* [15], the cumulative abundance of all ASVs from these two genera were calculated. Significant differences between treatments were assessed using Wilcoxon sum rank test or Dunn’s test, with adjustments made using Bonferroni post hoc method [36]. After the analysis of the dominant and most abundant bacterial taxa, the non-core microbiome was examined separately. For this purpose, the two more abundant AVSs, namely ASV1 and ASV2, were excluded, and the remaining ASVs were analyzed to assess potential differences in composition among developmental stages and host plants using Wilcoxon sum rank test or Dunn’s test with Bonferroni corrections for pairwise comparisons [36]. The same tests were then performed to assess differences in the abundance of ASVs belonging to the non-core microbiome, which was obtained after removing the two dominant ASVs. The 35 most abundant ASVs were visualized through a heatmap, created using log2 transformed mean abundances for developmental stages and host plants with ‘ComplexHeatmap’ package (version 2.26.0) [37]. Statistical differences among groups were assessed using the Dunn’s test, with adjustments made using Bonferroni post hoc method, and significantly different ASVs (adjusted *p* < 0.05) were marked with red asterisks on the heatmap.

## 3. Results

### 3.1. Metabarcoding Analysis of the Nymph Bacterial Communities

#### 3.1.1. Full Microbiome

After 16S rRNA gene metabarcoding of *S. titanus* nymphs, a dataset was obtained yielding 6,843,795 high-quality reads, with an average of 78,664 reads per sample (range: 42,414–126,582), which were clustered into 547 ASVs (project accession number PRJNA1321563).

**Figure 1:**
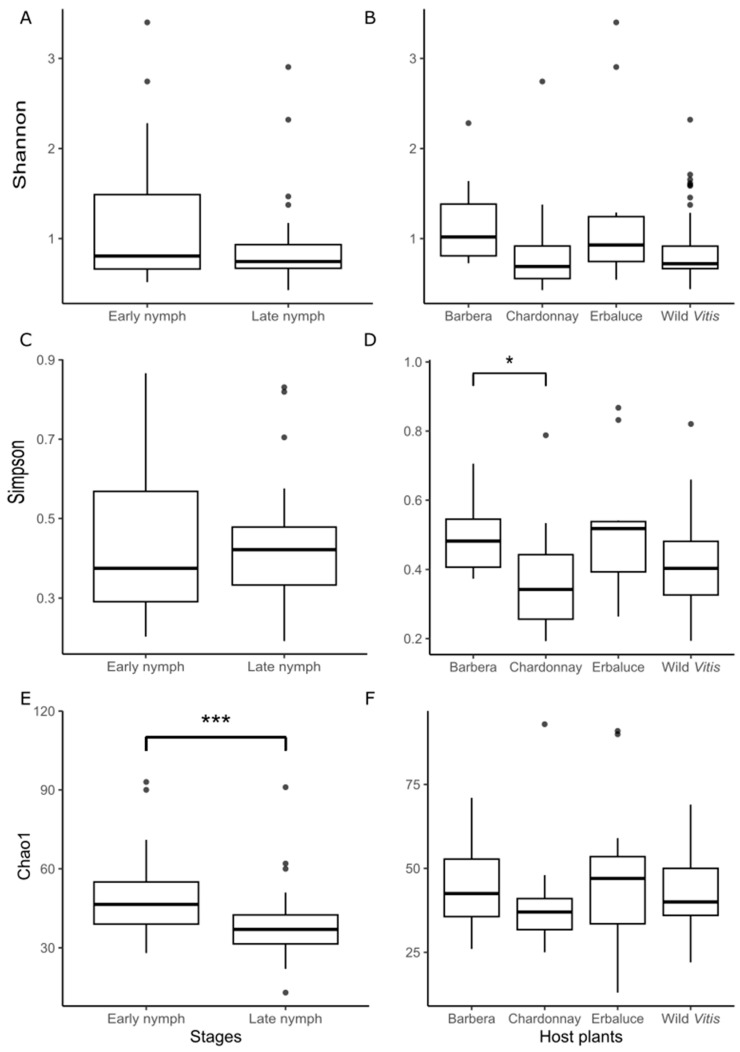
Alpha diversity indices of the microbiome of *Scaphoideus titanus* early and late nymphs reared on different host plants. Shannon (**A**,**B**), Simpson (**C**,**D**) and Chao1 (**E**,**F**) are shown for comparisons by developmental stages (**A**,**C**,**E**) and host plants (**B**,**D**,**F**). In (**D**), the asterisk indicates a significant difference in the pairwise comparisons by Dunn–Kruskal–Wallis test (* *p* < 0.05), whereas in (**E**) asterisks indicate significant differences in Wilcoxon rank sum test (*** *p* < 0.001).

*Alpha diversity*. Significant differences in alpha diversity were observed among treatments (Figure 1). Overall differences in the Shannon index were significant among nymphs reared on different host plants (Kruskal–Wallis χ^2^ = 8.92, df = 3, *p* = 0.030). However, post hoc Dunn’s test with Bonferroni correction revealed a marginally non-significant increase in diversity in individuals from Barbera compared to Chardonnay (Z = 2.62, unadjusted *p* = 0.0087, adjusted *p* = 0.052, Figure 1B). No other pairwise comparisons were significant (Figure 1B), including those between developmental stages (Wilcoxon rank-sum test, W = 1054, *p* = 0.243, Figure 1A). Considering the Simpson index, a significant effect of the host plant was detected (Kruskal–Wallis χ^2^ = 10.6, df = 3, *p* = 0.014). Dunn’s test indicated a significantly higher diversity in Barbera compared to Chardonnay (Z = 2.82, adjusted *p* = 0.029), whereas the comparison between Chardonnay and Erbaluce approached significance (Z = −2.49, adjusted *p* = 0.076) (Figure 1D). Again, no significant differences in alpha diversity between developmental stages were detected (W = 903, *p* = 0.901, Figure 1C). In contrast, Chao1 richness did not differ significantly among host plants (Kruskal–Wallis χ^2^ = 3.86, df = 3, *p* = 0.28, Figure 1F), but it was significantly higher in early nymphs than in late nymphs, as revealed by the Wilcoxon rank-sum test (W = 1323.5, *p* = 0.00048) (Figure 1E).

**Figure 2:**
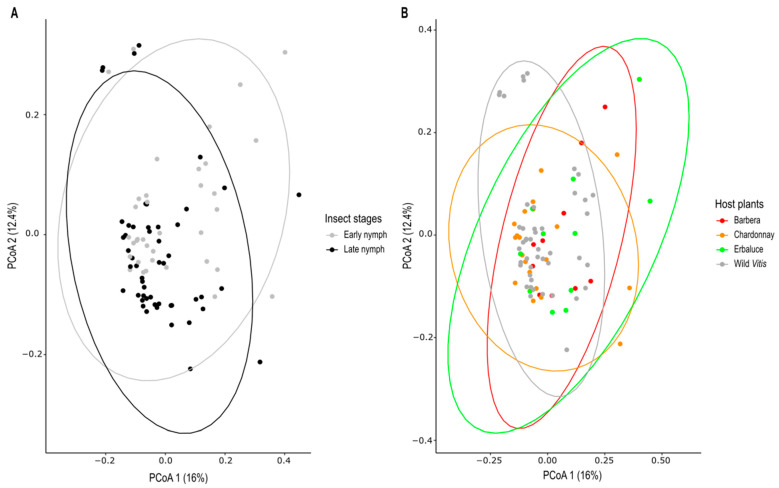
Beta diversity of *Scaphoideus titanus* early and late nymphs reared on different host plants. PCoA based on Bray–Curtis dissimilarity matrix, with Hellinger-transformed ASV abundance table, is shown. In (**A**) the colour was assigned to different insect stage, with early nymphs in grey and late nymphs in black, while in (**B**) different colours refer to the host plant: Barbera (red), Chardonnay (orange), Erbaluce (green), and wild *Vitis* (grey).

*Beta diversity*. PERMANOVA was conducted to test the effects of host plant, developmental stage, and their interaction on the bacterial community compositions, revealing significant effects of both host plant (R^2^ = 0.0637, F = 1.95, *p* = 0.0009) and developmental stage (R^2^ = 0.0338, F = 3.10, *p* = 0.0002). In contrast, the interaction was not statistically significant (R^2^ = 0.0411, F = 1.26, *p* = 0.1037). In addition, the test for homogeneity of multivariate dispersion (PERMDISP) indicated a significant difference in community dispersion between developmental stages (F = 4.27, *p* = 0.0419), while no significant differences were recorded in the dispersion of nymphs reared on different host plants (F = 0.2994, *p* = 0.8258). Principal coordinates analysis (PCoA) of Bray–Curtis dissimilarity showed a partial segregation between developmental stages (Figure 2A) and between insects reared on American *Vitis* and *V. vinifera*, irrespective of the cultivar (Figure 2B). However, the significant PERMDISP result for developmental stages indicates that caution should be exercised in interpreting the separation in Figure 2A, as it may partially reflect differences in within-group dispersion rather than solely centroid locations.

**Figure 3:**
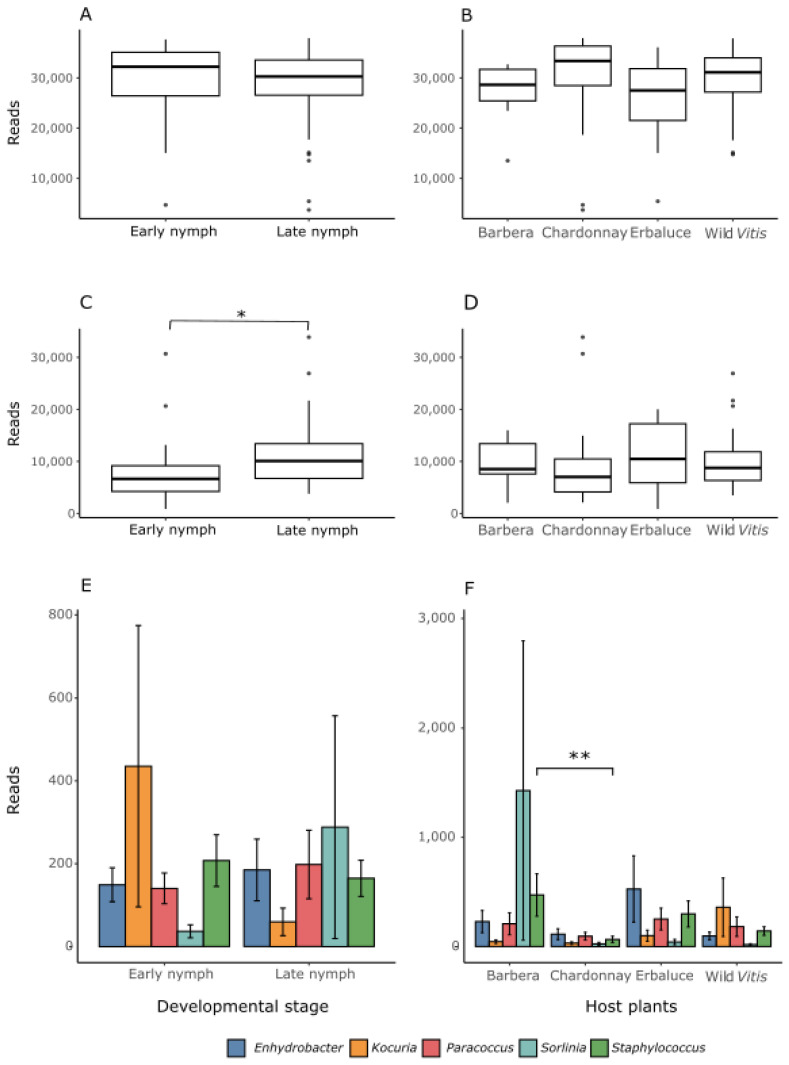
Relative abundance of the most abundant bacterial genera in *Scaphoideus titanus* early and late nymphs fed on different host plants. The relative abundance of *Karelsulcia* grouped by developmental stage and host plants are shown in (**A**,**B**), respectively. The relative abundance of *Cardinium* grouped by developmental stage and host plants are shown in (**C**,**D**), respectively. The relative abundance of the five more abundant genera after *Karelsulcia* and *Cardinium* is presented in function of developmental stage (**E**) and host plant (**F**). Bars represent the mean number of reads ± standard error for each taxon. Asterisks indicate significant differences after Wilcoxon rank sum test (* *p* < 0.05) or Dunn’test followed by pairwise comparisons adjusted by Bonferroni postdoc (** *p* < 0.01) in (**C**,**F**), respectively.

*Bacterial taxonomic analysis*. To describe the bacterial composition, a feature table was presented at the genus level in a taxonomy barplot (Figure S1). As expected, the bacterial community of *S. titanus* nymphs was predominantly composed of two symbiotic genera, corresponding to *Karelsulcia* and *Cardinium*, with 68.392% and 22.75% of the total reads assigned to the former and the latter, respectively. The abundances of the two dominant symbionts were compared among nymphs reared on different host plants and at different developmental stages (Figure 3A–D). The results of Wilcoxon rank-sum test comparing the relative abundance of *Karelsulcia* between developmental stages showed no statistically significant difference (W = 1019, *p* = 0.386). In contrast, the relative abundance of *Cardinium* differed significantly between developmental stages, as revealed by the Wilcoxon rank-sum test (W = 528, *p* = 0.00079), indicating an increase in late nymphs. When it comes to the host plant, the results of Kruskal–Wallis tests comparing the relative abundance of *Karelsulcia* showed slight non-significant fluctuations (χ^2^ = 6.60, df = 3, *p* = 0.086); similarly, no significant differences were detected for *Cardinium* (χ^2^ = 2.18, df = 3, *p* = 0.536). Beyond *Karelsulcia* and *Cardinium*, many of the most abundant ASVs were not assigned to any genus; therefore, their taxonomic position was explored using BLAST searches. The BLAST results showed that ASV14 (Unassigned Acetobacteriaceae) was identical to *Sorlinia euscelidii* (Acc. No. NR_199532); ASV17 (Unassigned Corynebacteriaceae) was identical to *Corynebacterium* sp. (Acc. No. ON786377); ASV32 (Unassigned Enterobacteriaceae) was identical to *Klebsiella pneumoniae* (CP191474); ASV18 (Unassigned Enterobacterales) showed 98.88% similarity to *Pantoea* sp. (Acc. No. MK480045). The remaining unassigned ASVs did not show high similarity (i.e., higher than 97%) with any described bacterial genus in BLAST; they were classified as unassigned genus.

The top five most abundant genera apart from *Karelsulcia* and *Cardinium* included *Enhydrobacter*, *Kocuria*, *Paracoccus*, *Sorlinia*, and *Staphylococcus*; their relative abundances were compared across host plants and developmental stages. Wilcoxon tests revealed no significant differences across developmental stages for any of these genera (Figure 3E). When considering the host plant effect, only *Staphylococcus* showed a significant difference in abundance among host plants (Kruskal–Wallis tests: χ^2^ = 13.9, df = 3, *p* = 0.004). Pairwise Dunn’s test indicated a higher abundance in insects feeding on *V. vinifera* cv. Barbera than in those feeding on Chardonnay (Z = 3.59, *p*.adj = 0.002) (Figure 3F).

A deeper investigation was performed considering the taxonomic attribution of the most abundant taxa. The genus *Karelsulcia* was represented by three distinct ASVs (ASV1, ASV448, ASV502) and *Cardinium* by five (ASV2, ASV3, ASV33, ASV116, ASV306). The single ASVs were not equally represented in the samples. ASV1 of *Karelsulcia* accounted for 68.386% of the total reads (99.99% of *Karelsulcia*-related reads), and ASV2 of *Cardinium* accounted for 20.99% of the total reads and 92.26% of reads belonging to this genus. Therefore, ASV1 and ASV2 were recognized as the two dominant ASVs in tested nymphs. Strikingly, in six nymphs reared on American *Vitis* that lacked ASV2, another *Cardinium*-related ASV (namely ASV3) was one of the dominant ones (25.43% of total reads and 99.94% of *Cardinium*-related reads in these specimens).

#### 3.1.2. Non-Core Microbiome

**Figure 4:**
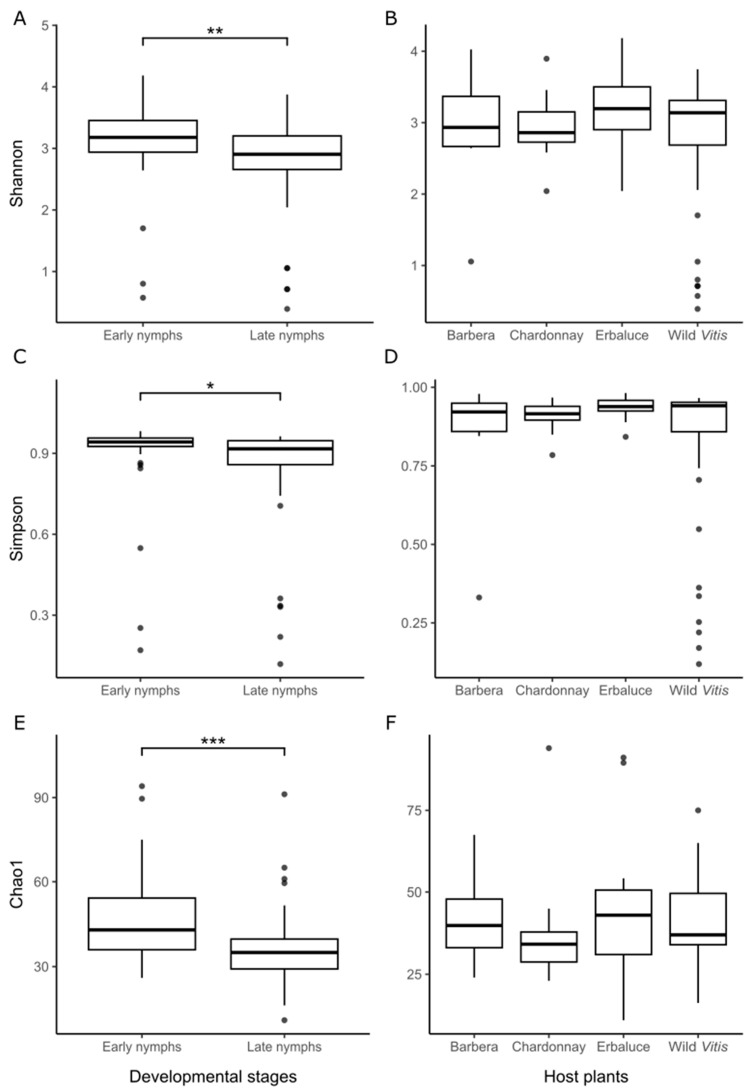
Alpha diversity indices of the non-core microbiome of *Scaphoideus titanus* early and late nymphs reared on different host plants. Shannon (**A**,**B**), Simpson (**C**,**D**) and Chao1 (**E**,**F**) are shown for comparisons by developmental stages (**A**,**C**,**E**) and host plants (**B**,**D**,**F**). Asterisks indicate significant differences in Wilcoxon rank sum test (* *p* < 0.05, ** *p* < 0.01, *** *p* < 0.001).

*Alpha diversity*. After removing the two dominant ASVs, associated with *Karelsulcia* (ASV1) and *Cardinium* (ASV2), alpha diversity metrics were recalculated to characterize the non-core microbiome. A significant difference in all indices was detected between developmental stages (Shannon: Wilcoxon W = 1265, *p* = 0.0028, Figure 4A; Simpson: Wilcoxon W = 1206, *p* = 0.0132, Figure 4C; Chao 1: Wilcoxon W = 1314, *p* = 0.00065, Figure 4E), with lower diversity in late nymphs, whereas no significant differences were observed among nymphs reared on different host plants (Shannon: Kruskal–Wallis χ^2^ = 3.16, df = 3, *p* = 0.367, Figure 4B; Simpson: Kruskal–Wallis χ^2^ = 2.73, df = 3, *p* = 0.436, Figure 4D; Chao1: Kruskal–Wallis χ^2^ = 3.95, df = 3, *p* = 0.267, Figure 4F).

**Figure 5:**
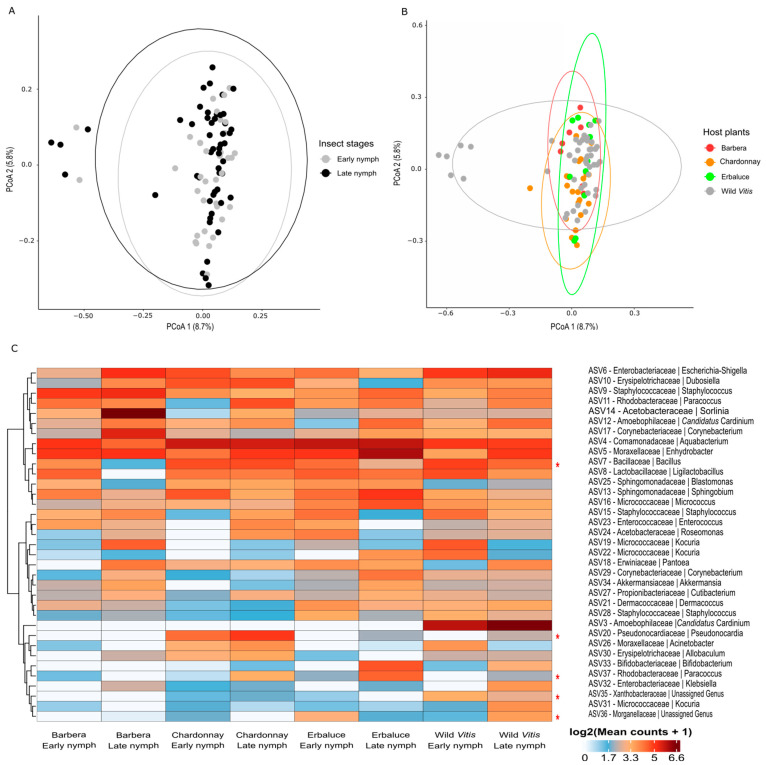
Beta diversity and taxonomic composition of the non-core microbiome of *Scaphoideus titanus* nymphs. PCoA based on Bray–Curtis dissimilarity matrix after Hellinger transformation is shown in (**A**) (as a function of the developmental stages) and (**B**) (as a function of the host plant). The heatmap in (**C**) shows the average log_2_-transformed relative abundances of the 35 most abundant ASVs, after removal of ASV1 and ASV2, in *S. titanus* nymphs grouped by developmental stage and host plant, clustered by Euclidean distances. Asterisks show significant differences for the indicated ASV.

*Beta diversity*. After the removal of ASV1 and ASV2, PERMANOVA confirmed significant effects of the host plants (F = 1.92, R^2^ = 0.064, *p* < 0.001) and developmental stage (F = 1.69, R^2^ = 0.019, *p* = 0.002) on the bacterial composition, whereas their interaction was not statistically significant (F = 1.03, R^2^ = 0.035, *p* = 0.364). In contrast, the beta-dispersion test that was conducted to evaluate the homogeneity of multivariate dispersions across groups did not reveal any significant effect of either the developmental stage (F = 0.15, *p* = 0.697) or the host plants (F = 0.56, *p* = 0.645), indicating that the observed PERMANOVA effects were likely driven by differences in group centroids rather than within-group variability. Interestingly, the PCoA showed a segregation that was not explained by the developmental stage (Figure 5A) but corresponded to six nymphs reared on American *Vitis* (Figure 5B). The separated specimens were the six nymphs hosting ASV3 as the dominant *Cardinium* and lacking ASV2 (Figure S2).

*Bacterial taxonomic analysis*. After removing the two dominant symbionts from the dataset, statistical analyses revealed significant differences in the relative abundance of five bacterial ASVs across the host plants-developmental stages combinations (Figure 5C). ASV37 (*Paracoccus*) exhibited significantly diverging relative abundances observed among several host-stages combinations. The highest relative abundance was recorded in late nymphs reared on Erbaluce, which was significantly higher than in early nymphs reared on wild *Vitis* (*p* < 0.001, Z = 4.75), late nymphs reared on Chardonnay (*p* = 0.001, Z = −4.07), late nymphs reared on Barbera (*p* = 0.002, Z = −3.81), and late nymphs reared on wild *Vitis* (*p* = 0.008, Z = 3.45). ASV20 (*Pseudonocardia*) also showed significant abundance differences, with the highest relative abundance found in late nymph reared on Chardonnay. This group differed significantly from early nymphs reared on wild *Vitis* (*p* < 0.001, Z = 4.44), early nymphs reared on Erbaluce (*p* = 0.046, Z = 2.94), and late nymphs reared on Barbera (*p* = 0.023, Z = −3.14). ASV7 (*Bacillus*) exhibited significant differences in relative abundance between late nymphs reared on Barbera and both early nymphs reared on wild *Vitis* (*p* = 0.009, Z = −3.41) and late nymphs reared on Chardonnay (*p* = 0.050, Z = −2.92). ASV36 (unassigned Morganellaceae) varied significantly between early nymphs reared on Erbaluce and both late nymphs reared on Chardonnay (*p* = 0.010, Z = −3.38) and late nymphs reared on wild *Vitis* (*p* = 0.012, Z = 3.34). Finally, ASV35 (unassigned Xanthobacteraceae) differed significantly between early nymphs reared on wild *Vitis* and both late nymphs reared on Barbera and late nymphs reared on Erbaluce (both *p* = 0.032, Z = −3.05). The only ASV showing total segregation was ASV3 (*Cardinium*), which was exclusively found in nymphs reared on wild *Vitis*.

## 4. Discussion

The close relationship between *S. titanus* and *V. vinifera* is a typical trait of the insect populations that have invaded Europe, whereas in its native Nearctic range the leafhopper prefers *V. labrusca* and *V. riparia* [38]. A preference for American *Vitis* spp. over *V. vinifera* has been documented by field observations [19], although multiple parameters may influence the difference in *S. titanus* field abundance between cultivated and wild areas, such as insecticide treatments in vineyards. The ability of *S. titanus* to thrive in different environments and feed on alternative hosts has significant implications for its role as a vector of FD phytoplasma. Previous studies have shown that *S. titanus* can colonize vineyards late in the season after developing on surrounding wild or cultivated *Vitis* species. This increases the risk of phytoplasma transmission in later vine phenological stages [15]. Furthermore, *S. titanus* can acquire the phytoplasma relatively quickly in the adult stage and transmit it to cultivated vines, which highlights the importance of non-cultivated host plants as reservoirs of infection [39], emphasizing the pivotal role of landscape management in controlling the spread of FD by monitoring alternative host plants and implementing targeted control strategies.

Even though previous observations showed a significant effect of the host plant on the development of *S. titanus* nymphs [22], the host plant had a rather moderate impact on the *S. titanus* full microbiome, as no difference was found in the alpha diversity indices of nymphs fed on different *Vitis* species. Only the comparison between Barbera and Chardonnay cultivars within *V. vinifera* yielded a significant difference, especially in the Simpson index. Nymphs reared on Chardonnay showed indeed the lowest diversity, suggesting higher dominance of few ASVs than in the other insects. Accordingly, these nymphs hosted the highest relative abundance of the primary symbiont *Karelsulcia*, corroborating its marked dominance within the microbiome of Chardonnay-reared nymphs. The difference with nymphs reared on Barbera may be due to possible variation in the phloem composition. It has been reported that lower amino acid content in grapevine phloem does not affect the fitness of *S. titanus* [40]; possible limited availability of some of the essential amino acids in the phloem of the offered Chardonnay plants may be compensated by higher abundance of *Karelsulcia*, whose main function is amino acid provisioning to the host [2]. Consistently with the high dominance of *Karelsulcia* in Chardonnay-reared insects, these samples showed a lower relative abundance of other ASV, especially *Staphyloccocus*.

Beta diversity analysis indicated a partial segregation of the bacterial communities of insects fed on different hosts. However, this was mainly due to few diverging specimens within each group, whereas the microbiomes of individuals reared on different *V. vinifera* cultivars and on American *Vitis* largely overlapped, as shown by PCoA based on Bray–Curtis dissimilarities, which was corroborated by the multivariate dispersion test. This suggests that the microbial composition is shaped by multiple interacting factors, including individual variability, plant-related traits and ontogenetic changes, with a generally comparable level of community heterogeneity across conditions.

Considering the life stage progression, a mild effect on microbiome alpha diversity was observed regardless of the host plant, with late nymphs showing only a significantly lower Chao 1 index compared to early nymphs. This indicates a reduction in the contribution of rare ASVs to the microbiome during development. This aligns with the identification of *Karelsulcia* and *Cardinium* ASVs as the dominant bacteria in all populations and stages, corroborating previous studies on the *S. titanus* microbiome [17,18]. As expected, given that they are reported to be vertically transmitted in their hosts [2,41,42], both symbionts were detected at all stages. The dominant condition was expected for *Karelsulcia*, which is widely reported to be the primary symbiont of Auchenorrhyncha and to provide hosts with essential amino acids to complement their diet [43]. The exact function of *Cardinium* in *S. titanus* remains unclear; however, several studies indicate this symbiont acts as a reproductive manipulator inducing various phenotypic effects in different hosts [44,45,46]. Reproductive manipulation could explain the near-perfect prevalence of *Cardinium* infection in *S. titanus*, although an additional mutualistic role cannot be ruled out.

The relative abundance of the two dominant symbionts tended to increase at the late nymph stages. Nevertheless, the increase was significant only for *Cardinium*, suggesting that this symbiont may be a major driver of *S. titanus* microbiome diversity. The increase in the relative abundance of *Cardinium* may be the result of bacterial multiplication in the host body combined with possible horizontal transfer via co-feeding [47] among nymphs from the same replicate.

It should be noted that multiple ASVs were identified in *S. titanus* nymphs in this study for both *Karelsucia* and *Cardinium*, suggesting a higher diversity of these symbionts than was previously recognized [18]. However, only a few SNPs were found in the minor ASVs; therefore, it is unclear whether these can be attributed to real sequence variability or rather to sequencing errors, particularly when co-occurrence was recorded between ASVs related to the same symbiont in a single specimen. Furthermore, it remains unclear whether the putative variants do reflect distinct symbiont haplotypes or are multiple copies of the 16S gene from a single genotype. Future studies focusing on the genetic diversity of the dominant bacterial symbionts of *S. titanus* may also contribute to clarify their role in this leafhopper, particularly with regard to *Cardinium*. Interestingly, one *Cardinium*-related ASV (ASV3), distinct from the dominant ASV, was found in high concentrations in a few samples reared on American *Vitis*, irrespective of life stage. In these specimens, ASV3 was the only *Cardinium*-related sequence found, suggesting competitive replacement in insect colonization (e.g., through reproductive incompatibility). The implications for the host fitness of infection with this symbiont variant should be deeply investigated, as well as the intensity of its relationship with wild vines.

Excluding the two most abundant ASVs (attributed to *Karelsulcia* and *Cardinium*) revealed higher divergence in the non-core microbiome alpha diversity of nymphs at different stages, with all indices decreasing in the late instars. This lower diversity in late-stage nymphs suggests that the non-core microbiome is selected during post-embryonic development, with a relevant proportion of the acquired bacterial community being lost over time after ingestion. Conversely, no difference in alpha diversity was found in relation to the host plant, confirming that a similar trend occurs in nymphs regardless of the food source. Interestingly, a difference was found in beta diversity for almost all comparisons in this case, suggesting that both the post-embryonic development and the host plant affect the composition of the non-core microbiome. Therefore, although the abundance of the minor components of *S. titanus* microbiome decreases over time, the composition varies according to the plant offered, as also indicated by the significant divergence in the relative abundance of several minor ASVs. In this context, an important role seems to be played by *Cardinium* ASV3, as the specimens hosting this bacterium were clearly distinct from the others in the PCoA graph. The genetic variability of *Cardinium*, which may be related to symbiont incompatibility and competition within the host, is hence likely to play a significant role in determining differences in the microbiome composition. The modality by which the suggested competition occurs merits investigation, particularly given the apparent segregation of this association within the American *Vitis* population.

Under field conditions, where an insect may move to a different grape cultivar or species by flying at the adult stage, any variation may facilitate the movement of bacteria (including plant endophytes) from plant to plant. The suggested bacterial movement could be particularly relevant in the case of adult *S. titanus* flying from wild to cultivated vines [15,19,21], considering that American vines are regarded as a preferred host [38]. This is in addition to other agronomic factors, such as the absence of insecticide treatments outside vineyards, where wild vines are mainly found. The extent to which *S. titanus* can modulate the plant microbiome by transferring bacteria from wild to cultivate vines is currently unknown, as well as the possible effect on the plant health. However, significant local variations cannot be excluded. Moreover, the moderate yet significant microbial differentiation observed here supports the hypothesis that microbiome plasticity could contribute to confer a degree of ecological versatility upon *S. titanus*. If this hypothesis was confirmed, any microbiome divergence may affect leafhopper efficiency in transmitting FD phytoplasma [48]; therefore, additional studies are needed to further investigate this issue.

Taken together, our results increase our knowledge on the adaptation of *S. titanus* to different *Vitis* species and suggest a moderate involvement of plant microbiome in this process. The spread of FD in Europe is due to the efficient adaptation of a Palearctic phytoplasma to a Nearctic vector [16]. Interactions between the phytoplasma, the insect vector, and the host plant may not be driven only by genetic compatibility, but also by factors related to the microbiome of *S. titanus*. These factors may include facultative symbionts, such as *Cardinium*, which were acquired after the invasion of Europe and that may have favored certain insect genotypes, for example, through reproductive manipulation. Our results highlight the need for future investigations into how agroecosystem management in vineyards and surrounding areas could affect the insect microbiome, and whether this could mitigate FD spread.

## Data Availability

The sequence data presented in the study are openly available at the Project PRJNA1321563. All additional data are presented in the main text and Supplementary Materials. Further inquiries can be directed to the corresponding author.

## Supplementary Materials

The following supporting information can be downloaded at: https://www.mdpi.com/article/10.3390/insects16111144/s1, Figure S1: Taxonomic composition of *Scaphoideus titanus* microbiome at the genus level; Figure S2: Samples heatmap of the 35 most abundant ASVs after removal of ASV1 and ASV2. Dataset was log2 transformed and clustered by Euclidean distances.
