# Supplementary material for: Effects of Host Plant on the Bacterial Community of the Leafhopper Scaphoideus titanus
Source: Insects. 2025 Nov 8;16(11):1144. doi: 10.3390/insects16111144 (PMC12653582; doi:10.3390/insects16111144)
Supplement: Supplementary file 1 [file insects-16-01144-s001.zip › insects-3943872-supplementary.pdf]

insects

MDPI

# Effects of host plant on the bacterial community of the leafhopper *Scaphoideus titanus*

**Andrea Arpellino, Aya M.A. Elsayed, Elena Gonella* and Alberto Alma**

Department of Agricultural, Forest and Food Sciences, University of Torino, Italy
andrea.arpellino@unito.it
aya.elsayed@unito.it
elena.gonella@unito.it
alberto.alma@unito.it

* Correspondence: elena.gonella@unito.it Tel.:+390116708532

## Supplementary Materials

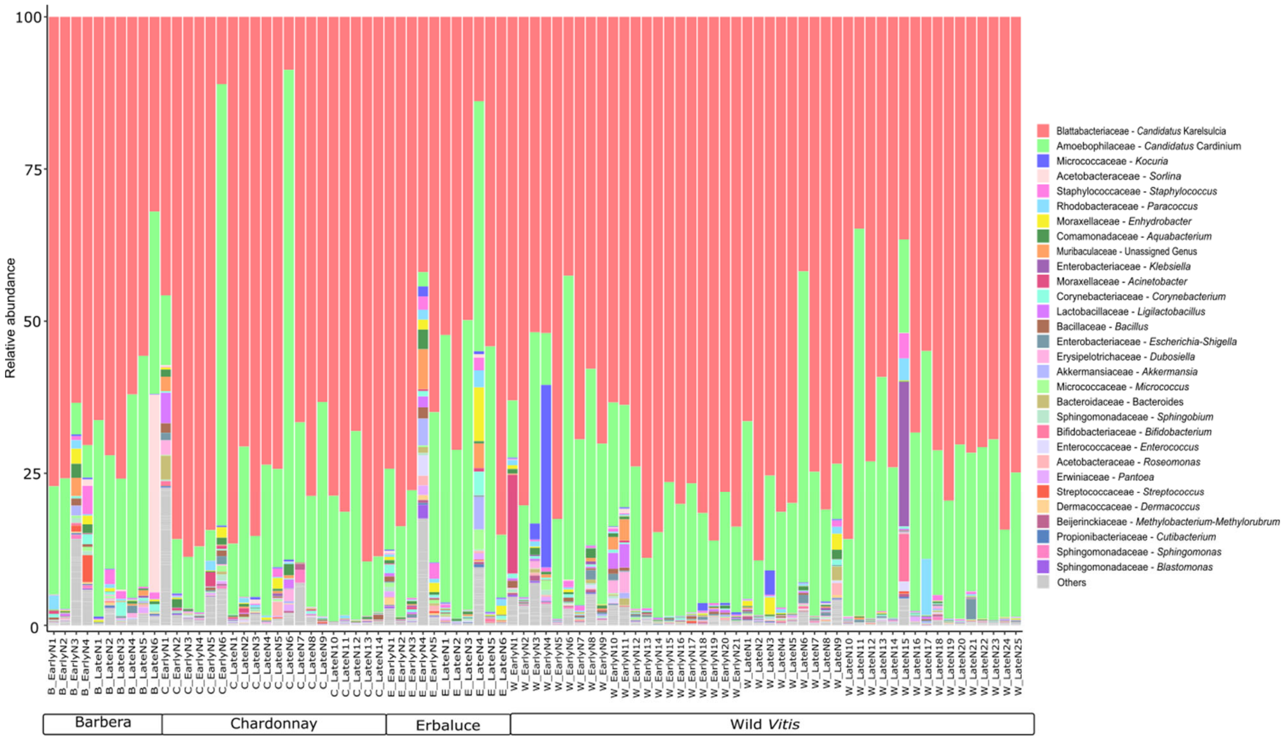


**Figure S1.** Taxonomic composition of *Scaphoideus titanus* microbiome at the genus level. Taxonomy barplot reporting the 30 most abundant bacterial genera in the microbiome of *S. titanus* nymphs.

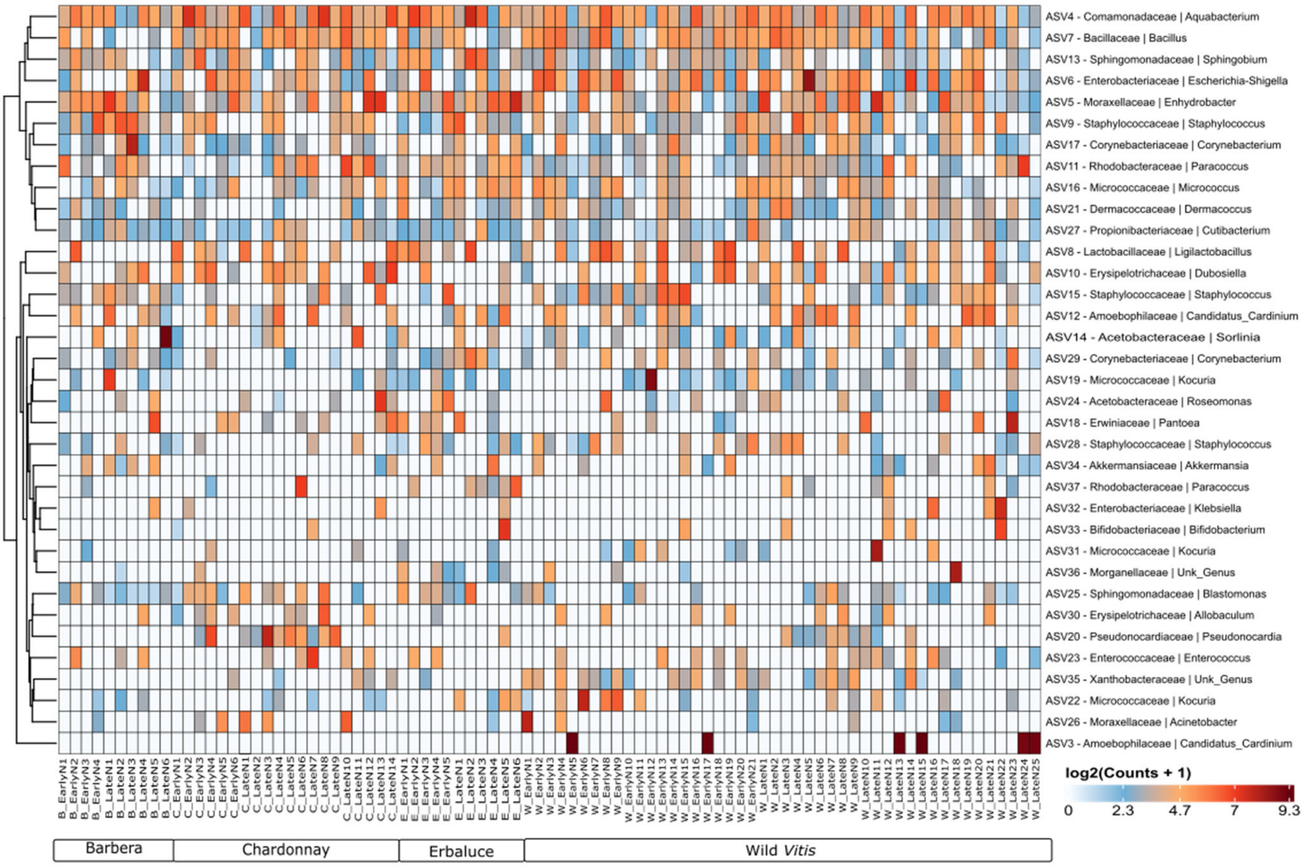


Fugure S2 Samples heatmap of the 35 most abundant ASVs after removal of ASV1 and ASV2. Dataset was log2 transformed and clustered by Euclidean distances.
